# Flavonoid Interaction with a Chitinase from Grape Berry Skin: Protein Identification and Modulation of the Enzymatic Activity

**DOI:** 10.3390/molecules21101300

**Published:** 2016-09-28

**Authors:** Antonio Filippi, Elisa Petrussa, Uros Rajcevic, Vladka Čurin Šerbec, Sabina Passamonti, Giovanni Renzone, Andrea Scaloni, Marco Zancani, Angelo Vianello, Enrico Braidot

**Affiliations:** 1 Department of Agricultural, Food, Animal and Environmental Sciences, University of Udine, Via delle Scienze 91, 33100 Udine, Italy; antonio.filippi@uniud.it (A.F.); elisa.petrussa@uniud.it (E.P.); marco.zancani@uniud.it (M.Z.); angelo.vianello@uniud.it (A.V.); 2 Blood Transfusion Centre of Slovenia, Department of R&D, Šlajmerjeva 6, 1000 Ljubljana, Slovenia; uros.rajcevic@ztm.si (U.R.); vladka.curin@ztm.si (V.Č.Š.); 3 Department of Life Sciences, University of Trieste, via L. Giorgieri 1, 34127 Trieste, Italy; spassamonti@units.it; 4 Proteomics and Mass Spectrometry Laboratory, ISPAAM, CNR, via Argine, 1085-80147 Napoli, Italy; giovanni.renzone@ispaam.cnr.it (G.R.); andrea.scaloni@ispaam.cnr.it (A.S.)

**Keywords:** catechin, chitinase, pathogenesis-related proteins, quercetin, *Vitis vinifera*

## Abstract

In the present study, an antibody raised against a peptide sequence of rat bilitranslocase (anti-peptide Ab) was tested on microsomal proteins obtained from red grape berry skin. Previously, this antibody had demonstrated to recognize plant membrane proteins associated with flavonoid binding and transport. Immuno-proteomic assays identified a number of proteins reacting with this particular antibody, suggesting that the flavonoid binding and interaction may be extended not only to carriers of these molecules, but also to enzymes with very different functions. One of these proteins is a pathogenesis-related (PR) class IV chitinase, whose in vitro chitinolytic activity was modulated by two of the most representative flavonoids of grape, quercetin and catechin, as assessed by both spectrophotometric and fluorimetric assays in grape microsomes and commercial enzyme preparations. The effect of these flavonoids on the catalysis and its kinetic parameters was also evaluated, evidencing that they determine a hormetic dose-dependent response. These results highlight the importance of flavonoids not only as antioxidants or antimicrobial effectors, but also as modulators of plant growth and stress response. Implications of the present suggestion are here discussed in the light of environment and pesticide-reduction concerns.

## 1. Introduction

Flavonoids are a group of plant polyphenolic secondary metabolites, including red to purple anthocyanins, colourless to pale yellow flavonols (e.g., quercetin: QC), colourless to brown flavanols (e.g., catechin: CA) and proanthocyanidins or condensed tannins [1,2]. They are involved in several physiological functions, such as antioxidant activity, UV-light protection and defence against bacterial and fungal phytopathogens [3]. The latter function is related to some specific activities, such as: (i) the polyphenol oxidase polymerizing activity on sinapyl and coniferyl derivatives, which generates a physical barrier to pathogen invasion [4]; (ii) the inhibitory properties against essential enzymes for pathogens, as in the case of tannins; iii) the direct antimicrobial/antifungal action, as in the case of phytoalexins [5]. The antibiotic activity of flavonoids and of their brown derivatives may also depend, in part, on their antioxidant properties, either by acting as free radical scavengers or by preventing their formation by chelating metals.

To exert antibiotic action, flavonoids have to be either transported from the source tissue to the site of infection or increasingly produced by infected cells, where they induce the hypersensitive reaction against biotrophic pathogens, leading to a fast and localized programmed cell death [6]. This response is particularly important during fruit ripening, because such organ accumulates sugars and attractive molecules, so becoming more prone to biotic attack. Thus, plant cells start to synthesize pathogenesis-related (PR) proteins and flavonoids, whose efficiency seems to depend on the activation rate of the corresponding biosynthetic pathways [7], as shown by the finding that polyphenols exert antifungal activity, so inducing resistance [7].

Regarding the general ability of flavonoids to interact with proteins, relative binding properties and effect on the corresponding enzyme activities may depend on their molecular structure and oxidation state. In this context, examples have been reported for different enzymes, including NADH oxidases, polyphenol oxidases and peroxidases [4], lipoxygenases [8], cellulases, xylanases, pectinases, glutathione-*S*-transferases (GST) and glycoproteins and protein kinases involved in the polar auxin transport [6]. Indeed QC and kaempferol could inhibit the activity of auxin transport proteins through the interaction of their catechol group in the B-ring of the flavonoid skeleton [2,9].

Grapevine (*Vitis vinifera* L.) is one of the richest sources of polyphenols among fruits, and it is a common food in the human diet. Flavonoids are the most abundant phytonutrients with biological activity; actually, they possess cardio-protective, neuro-protective, antimicrobial and anti-aging properties [10]. Most flavonoids are found primarily in the outer epidermal cells of red grape skin, whereas ca. 60%–70% of total polyphenols are stored in seeds. Flavanols, one of the most abundant class of flavonoids found in grapevine, are present mainly in the form of (+)-CA, (−)-epicatechin, and proanthocyanidins [11]. In white grape varieties, flavanols represent 46%–56% of total phenolics, whereas in red grapes their concentration is in the 13%–30% range. Although flavonols are present only as 3-*O*-glycosides in grape skin, they can be found also as aglycones (QC, kaempferol, myricetin and isorhamnetin) in wines and juices, as a result of acid hydrolysis during their processing and storage. Although the profile of flavonols strongly depends on grape cultivars, QC, kaempferol and isorhamnetin derivatives are found in both red and white grapes [12].

Due to their extensive cultivation, grapevine varieties are sensitive to a great number of pathogens. These infections provoke heavy damages and yield losses, finally affecting wine quality. The spread of diseases are generally controlled by the application of chemical pesticides. To limit environment pollution, alternative strategies involve the activation of plant defence mechanisms by natural elicitors. This type of resistance, characterized by a systemic accumulation of PR-proteins, is mainly associated with the induction of systemic acquired resistance (SAR) induced by pathogens [13]. This resistance to biotic stress is generally based on multiple biochemical factors and involves PR-proteins (chitinases, peroxidases, and β-1,3-glucanases), several elicitor-induced defence responses (e.g., the lignification of cell walls), and production of flavonoid phytoalexins [14] that inhibit fungi growth [15,16].

In this complex scenario, the complete role of flavonoids is still under study, because they are both ubiquitous in plant cells and involved in several biological activities [10]. Depending on their multiple physiological roles and localization, flavonoids need to be efficiently transported to short- and long-distance sites. A number of transporters with this putative function have been identified, which may perform both facilitated-passive and active transport, sometimes overlapping in a synergistic manner. In particular, active transport seems to involve different enzymes, such as ABC (ATP-binding cassette) proteins, MATE (multidrug and toxic extrusion) proteins, GST and a bilitranslocase-like protein [17]. The latter protein was assigned on the basis of its recognition by an antibody raised against a peptide sequence of rat bilitranslocase (anti-peptide Ab) [18]. This study was undertaken to further characterize the nature of grape berry skin proteins reacting with anti-peptide Ab, and in particular the putative modulation of flavonoids on their enzymatic activities. The analysis could provide further information on the regulative activities exerted by flavonoids on plant cell metabolism, in particular on those pathways involved in biotic stress responses.

## 2. Results

### 2.1. Western Blot on Microsomal Fraction from Red Grape Skin

**Figure 1:**
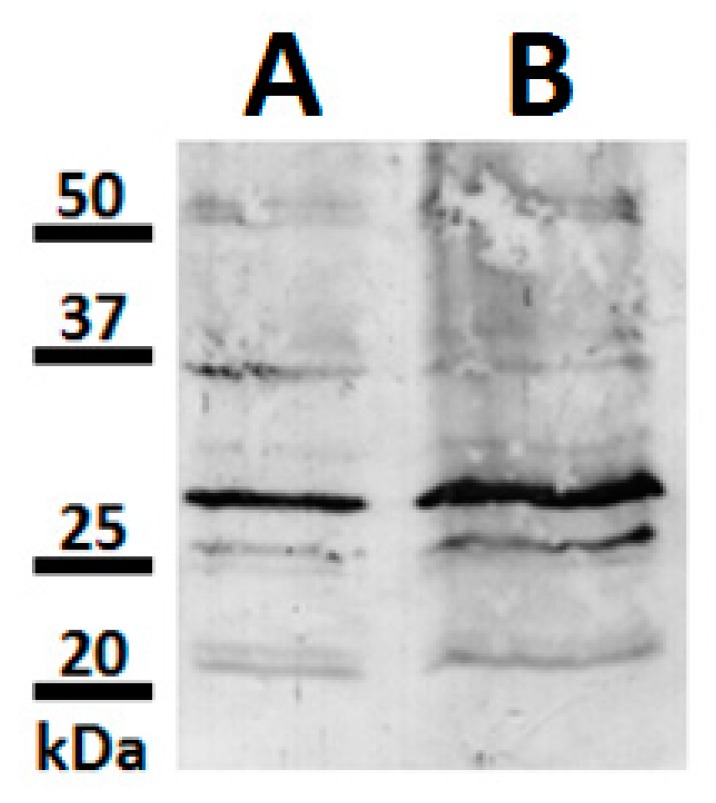
WB of proteins from RGSM revealing candidates for anti-peptide Ab interaction. RGSM proteins from cv. Merlot (15 and 30 µg, (**A**) and (**B**) respectively) were loaded onto 12% polyacrylamide gel. Western blotting was performed using mouse anti-peptide Ab at the concentration of 5 µg·mL^−1^. Anti-mouse IgM (dilution 1:15,000) was used as secondary antibody. Protein molecular mass markers are shown on the left.

Antibodies produced against the peptide 235–246 (EFTYQLTSSPTC) from bilitranslocase have been previously used to detect flavonoid membrane transporters in both human and plant materials [17,19]. In this context, preliminary experiments were performed on pulp and skin of grape fruits aimed at identifying putative flavonoid-binding proteins [17]. To further investigate this issue in grape, we used a novel mouse antibody (Ab), generated according to the method described before [20], which was utilized to assay grape skin microsomes. Western blot (WB) experiments demonstrated the presence of at least 5 bands migrating at 22, 25, 27, 37 and 50 kDa, respectively (Figure 1). We focused on the bands in the mass range of 25–27 kDa, since a quantitative correlation between signal intensity and protein concentration was obtained (A and B for 15 and 30 µg protein, respectively).

### 2.2. Proteomic Analysis

Two protein bands occurring in the range of 25–27 kDa were subjected to an integrated proteomic approach based on combined SDS-PAGE, 2-DE, WB and nLC-ESI-LIT-MS/MS analysis. These proteins were preliminarily purified by SDS-PAGE, thus reducing the complexity of the plant samples. Their resolution by isoelectric focusing within the 3.0–11.0 pH range allowed for a better visualization of the protein components recognized by the anti-peptide Ab. At the same time, it limited the occurrence of contaminants in the gel spots to be further subjected to mass spectrometric analysis for protein identification.

**Figure 2:**
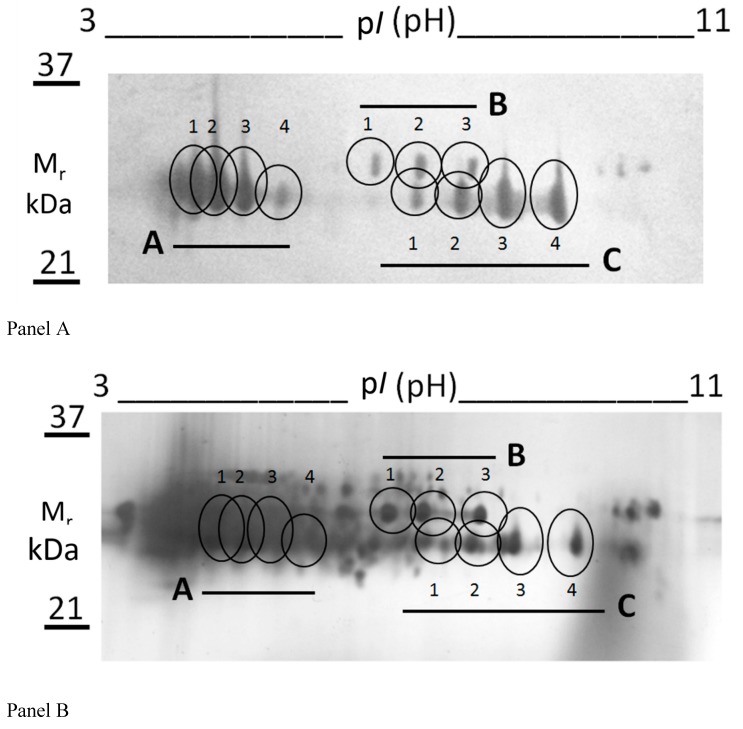
Immuno-proteomic analysis of grape skin proteins with a mass in the range 25–27 kDa. Thirty µg of RGSM proteins with a mass 25–27 kDa (Figure 1) was extracted from SDS-PAGE and subjected to 2-D electrophoresis, as described in the Materials and Methods section. Gels run in parallel were subjected to WB with anti-peptide Ab (Panel A) or to silver staining (Panel B). The “trains” of spots showing immuno-reactive signals were matched to counterparts in the silver-stained gel, and are labelled as A1-4, B1-3 and C1-4 in both panels. Spots of interest were digested with trypsin and subjected to MS analysis.

Figure 2 shows two representative gel images resulting from WB (Panel A) and silver nitrate staining (Panel B) of gel of the same protein sample after its preliminary purification by SDS-PAGE. A number of protein spots (A,B,C with numbering 1–4) was evident after WB with the anti-peptide Ab (Figure 2A); their counterparts within the silver nitrate stained gel were identified by software-assisted comparison of the corresponding 2-D images (Figure 2B). The limited number of spots detected by WB, with respect to that visualized by silver staining, demonstrated that only a very small portion of the proteins present within the samples was recognized by Ab (Figure 2).

In particular, they consisted in a “train” of four spots (spots A1-4) migrating in the pI range 4.0–5.5 and with an apparent mass of 25 kDa, a “train” of four spots (spots C1-4) migrating in the pI range 6.0–8.0 and with an apparent mass of 25 kDa, and a “train” of three spots (spots B1-3) migrating in the pI range 6.0–7.0 and with an apparent mass of 27 kDa. These spots were further digested with trypsin and the resulting digests were analyzed by nLC-ESI-LIT-MS/MS. The results of database searching are reported in Table 1.

Spots A1-4 and B1-3 corresponded to class IV chitinase (Q7XAU6) and glucan endo-1,3-β-glucosidase (XP_002277446), respectively. Otherwise, spots C1-4 contained a mixture of vicilin-like antimicrobial peptides 2-3 (XP_003632318) and triose phosphate isomerase (CAN70587) that migrated together within the gel as result of their almost identical pI and mass values. The observation that the theoretical mass of vicilin-like antimicrobial peptides 2-3 is 97 kDa suggested that a fragment of this protein was identified in this study.

In order to rationalize the reaction of the above-mentioned proteins with the anti-peptide Ab, in silico sequence alignment of class IV chitinase, glucan endo-1,3-β-glucosidase, vicilin-like antimicrobial peptides 2-3, and triose phosphate isomerase with the peptide 235-246 of rat bilitranslocase was performed by using the L-Align program. These alignments provided evidence that all these proteins contain certain sequence similarities with that of the peptide 235-246 (Table 2) used to generate the anti-peptide Ab.

In fact, alignments exhibited an acceptable Eigen value, which was always under the limit of 0.5; it is also noteworthy the constant occurrence of Q, S and P residues at positions 5, 9 and 10, respectively. Furthermore, the structural analysis of the tertiary structure of chitinase C from *S. griseus* (pdb 1WVU_A), which shares 69.4% identity and 83.8% similarity with chitinase from *S. griseus* (WP_044369170) and 41.0% identity and 74.2% similarity with class IV chitinase protein from grape (Q7XAU6), showed that the putative sequence recognized by the anti-peptide Ab is located at C-terminus of the protein and exposed to the aqueous environment.

### 2.3. Reaction of a Chitinase from S. griseus with Anti-Peptide Ab

**Figure 3:**
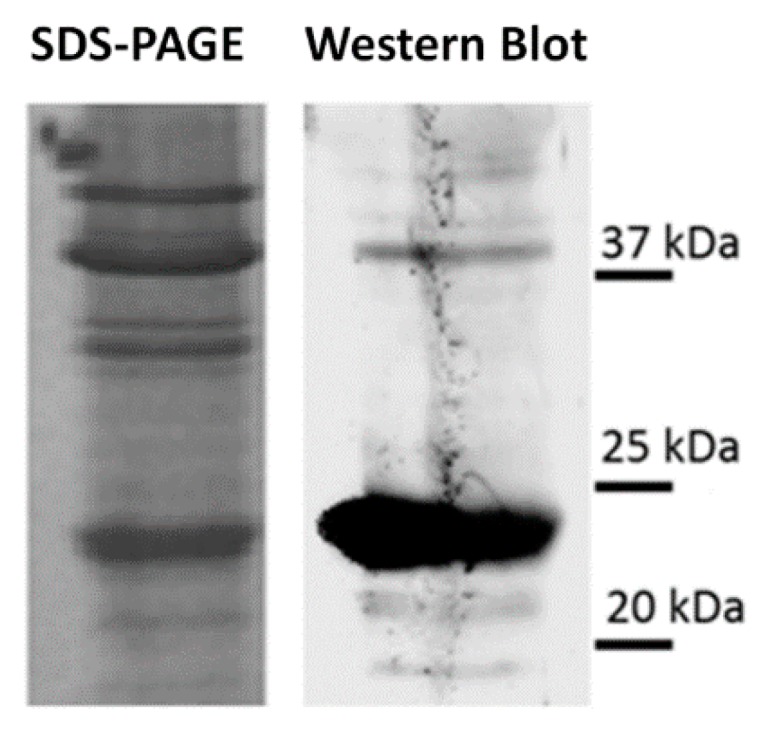
Reaction of a chitinase from *S. griseus* with anti-peptide Ab. Three micrograms of *S. griseus* chitinase was loaded onto 12% SDS-PAGE. Monoclonal anti-peptide Ab (5 μg IgM·mL^−1^) was used to detect the reaction with *S. griseus* chitinase. A Coomassie-stained gel corresponding to the counterpart challenged with Ab is also shown for comparison. Protein molecular mass markers are shown on the right.

With the aim of investigating whether the anti-peptide Ab was able to recognize a chitinase from an alternative source, a WB experiment was performed using a commercial enzyme from *S. griseus*. In this case, notwithstanding the low purification grade of the enzyme preparation, as evidenced by Coomassie-stained gel (Figure 3), the immuno-reaction showed a strong band, with an apparent mass of about 23 kDa.

This mass value was slightly lower than that predicted on the basis of the protein sequence and that measured in the case of red grape skin microsomes (RGSM) samples (Figure 1); accordingly, the enzyme was ascribed to family 19 bacterial chitinase C. This subtle difference may be due to the different mass value of the two chitinases, based on their amino acid sequence, or the occurrence of still unknown post-translational modifications. In this context, various enzymes belonging to the family of chitinases, with different mass values have already been identified in grape berries [21].

### 2.4. Acetazolamide Inhibition of Chitinase Activity

To further validate the occurrence of chitinase activity in berry skin extracts, samples of chitinase from RGSM and *S. griseus* were incubated with a specific inhibitor of this enzyme. Since various chitinase inhibitors of plant and animal chitinase isoforms have been described [22], acetazolamide was chosen, because of its effective inhibitory properties with both bacterial enzyme and plant extracts. Table 3 shows that the inhibitory effect of acetazolamide on the chitinase activity in RGSM extracts and *S. griseus* enzyme was 15.2% and 20.9%, respectively, confirming the presence of an active form of this enzyme in grape berry skin.

### 2.5. Inhibition of Chitinase Activity by Anti-Peptide Ab

To investigate the interaction between the anti-peptide Ab and the chitinase present in RGSM, the inhibitory effect of this antibody on the chitinase activity in RGSM extract and the *S. griseus* enzyme was assessed by two independent methods, which are based on absorbance [13] and fluorescence [23] measurements, respectively.

**Figure 4:**
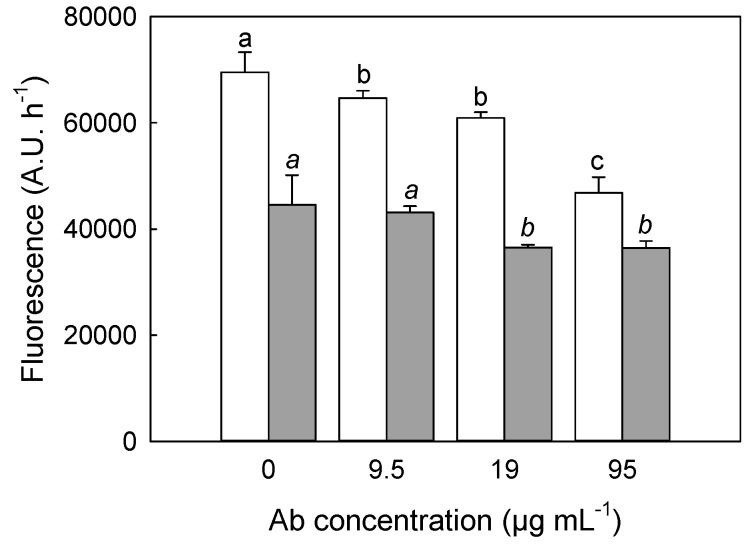
Inhibition of the chitinase activity of *S. griseus* and RGSM chitinase by anti-peptide Ab. Chitinolytic activity was measured as described in Schuttelkopf et al. [23]. 0.2 µg·µL^−1^ of *S. griseus* chitinase (white bars) and 30 µg of RGSM (grey bars) were assayed in the presence of increasing concentrations of anti-peptide Ab. Different letters assigned to means designate a statistical difference regarding data from *S. griseus* (not italic letters), or from RGSM samples (italic letters).

Both assays showed similar results, being Ab inhibitory on both chitinase samples. Figure 4 shows the data obtained by the fluorimetric method, which was preferred because of its accuracy, sensitivity and simplicity. In particular, addition of increasing amounts of the anti-peptide Ab determined an inhibition of the *S. griseus* chitinase enzymatic activity. Accordingly, a direct effect of the Ab on the catalytic properties of the enzyme was hypothesized as result of its interaction with protein regions involved in catalysis. A similar inhibitory response was obtained in the case of RGSM extracts, although with a less pronounced extent.

### 2.6. Modulation of Chitinase Activity by QC and CA

Since the anti-peptide Ab, developed to recognize a flavonoid-binding domain of bilitranslocase, reacted with a class IV chitinase from grape berry skin, it could be argued that chitinase activity may be modulated by flavonoids. Thus, flavonoids belonging to flavonol and flavan-3-ol classes (QC and CA, respectively), were incubated at different concentrations with *S. griseus* chitinase in order to characterize their effect on the kinetic parameters (Table 4).

Commercial chitinase activity was evaluated using fluorimetric assay. The modulatory effect of QC in the range of 0–20 µM was bi-phasic (Table 4, left values). In the range 0–3 µM, both Vmax and KM increased; however, they decreased at higher concentrations up to 20 µM QC. The combined effect of this uncompetitive modulation is that the catalytic performance of chitinase improves at low concentrations of QC. In fact, the increased KM implies a wider range of substrate concentrations, where reaction rate increases linearly. In addition, the substrate-saturated enzyme has a higher Vmax than the control one.

**Figure 5:**
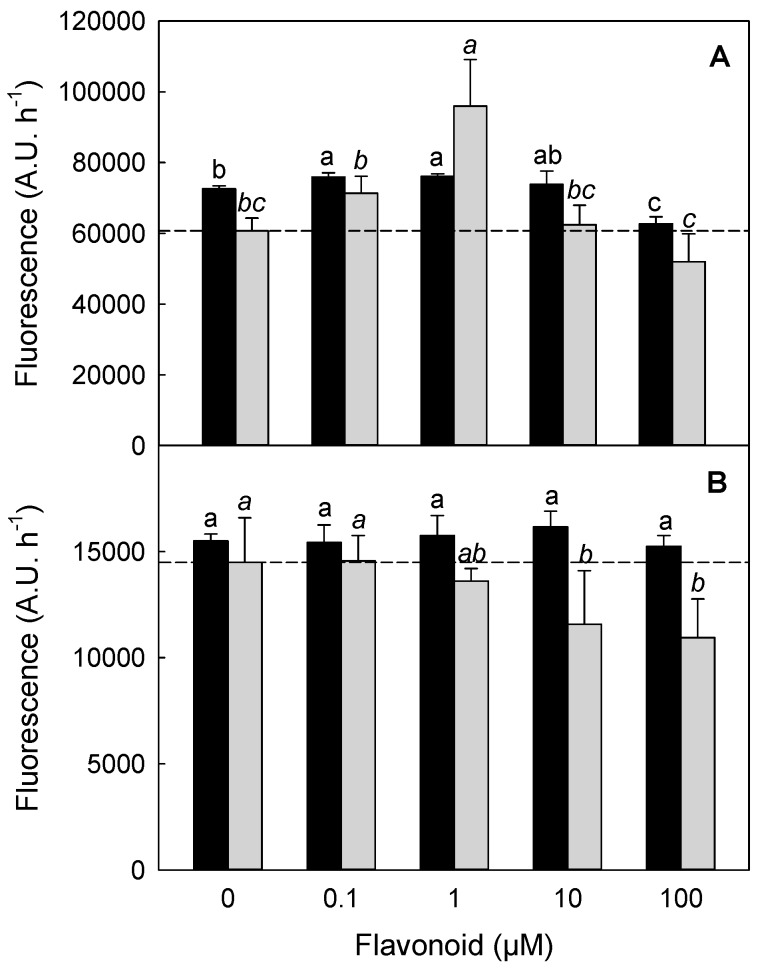
QC and CA modulation of the chitinase activity of *S. griseus* and RGSM enzyme. Chitinolytic activity was measured as described in Schuttelkopf et al. [23]. Different concentrations of CA (black bars) and QC (grey bars) were tested on chitinase from *S. griseus* (Panel A) and on RGSM (Panel B). Different letters assigned to means designate a statistical difference regarding data from CA (not italic letters), or from QC (italic letters).

Conversely, higher concentrations of QC reversed this gain of function, with a lower KM going along with a lower Vmax. A similar hormetic modulation was observed with CA (Table 4, right values). The peculiar response of commercial chitinase activity to increasing QC and CA concentrations was shown also in Figure 5 (panel A) and in supplementary Figure S1, where 0.1–100 µM range was used. CA (black bars) and QC (grey bars) showed a biphasic pattern described by a sort of hormetic dose-response effect. This phenomenon has already been reported in pharmacology, where low concentrations of a molecule exhibit an opposite effect on a certain enzymatic activity, when compared to high concentrations. Although the hormetic response was observed for both flavonoids, QC induced the strongest modulation; in particular, it determined a 60% increment of the chitinase activity when tested at 1 µM concentration, and a 20% decrement when assayed at 100 µM concentration (Figure 5, Panel A). At the same concentrations, CA stimulated the activity by about 5% and reduced it by almost 13%, respectively.

In the case of RGSM (Panel B), the effects of CA and QC were not as strong as in the case of *S. griseus* chitinase. In particular, no significant modulation was observed when RGSM were treated with CA. In contrast, an evident and linear inhibition was observed when QC was used. The apparent contrasting effect of CA and QC on the chitinase activity of RGSM, when compared to that of *S. griseus* enzyme, was rationalized hypothesizing the presence of endogenous flavonoids in the microsome extract, which might have masked and reduced the modulatory effect due to the external addition of CA and QC.

## 3. Discussion

Chitinases (EC 3.2.1.14) belong to a widely studied family of enzymes present in plant, animal and bacteria kingdoms [24]. These enzymes catalyze the hydrolytic cleavage of the β-1,4-glycosidic bond in *N*-acetylglucosamine-based biopolymers, mainly in chitin. Chitin is found in the cuticle of insect and crustacean shells, as well as in the cell walls of many fungi; accordingly, chitin is the second most abundant polysaccharide in nature after cellulose. Chitinases are the most studied PR-proteins, belonging to the families 18 and 19 of glycosyl hydrolases, which differ in amino acid sequence, structure and mechanism. While family 18 contains chitinases from many organisms, family 19 includes only highly conserved plant enzymes, typically endo-chitinases [22]. In bacteria, chitinases are mainly involved in nutrition processes; in yeast and various fungi, they participate in morphogenesis and some pathogenesis processes; in animals and plants, they mainly play a role in the defence against pathogen attack, as components of the innate immunity [25,26,27]. In the latter context, worth mentioning is the fact that different isoforms of chitinases can be induced by developmental signals regulating fruit ripening [21,28]. Specifically in the skin of grape berry, chitinase isoforms were over-expressed at maturation and the enzymatic activity correspondingly increased during ripening, even in the absence of any pathogen attack [28]. In grape berry from different cultivars, proteomic analysis evidenced that a class IV chitinase (CTG1027246) was strongly transcripted during post-veraison stage, where this PR protein is presumed to be involved in disease and pest resistance [29]. By generating or degrading signal metabolites, chitinases also participate to signalling pathways, including hormonal (ethylene) interaction [30]. This phenomenon is confirmed by the observation that chitinases and PR-proteins increase their activity during plant senescence [31]. Their involvement in programmed cell death has been hypothesized in *Arabidopis* and *Brassica napus* [30].

In the present work, a class IV chitinase (family 19) was identified in microsomes from red grape berry teguments as the main protein reacting with a mouse IgM Ab raised against the peptide 235-246 of rat bilitranslocase. The latter protein was chosen as a probe species being a flavonoid translocator [32]; peptide 235-246 was selected being present in the bilitranslocase region involved in the flavonoid binding [33]. Class IV chitinase identification was obtained by a combination of SDS-PAGE, 2-DE, WB and nLC-ESI-LIT-MS/MS experiments (Figure 2). Evidence of the existence of a protein similar to that identified here as reacting with this antibody derived from a previous work, where bilitranslocase-like proteins were characterized in grape berry skin microsomes by using rabbit IgG polyclonal Ab raised against the same peptide [18,19]. Although the immune-chemical assays were performed on similar plant extracts, the two antibodies exhibited a slightly different cross-reactivity. Mouse IgM Ab was able to cross-react with about five proteins (Figure 1), while rabbit IgG Ab detected only two polypeptides having similar molecular mass values (28–30 kDa). Mouse IgM Ab also reacted with a chitinase having bacterial origin (from *S. griseus*), which was used as a positive control (Figure 3). The latter is a bacterial member of the family 19 chitinases sharing sequence similarities with plant counterparts. In particular, family 19 chitinases are widely observed in the plant kingdom and, specifically, in grapevine [34], but they have also been characterized in bacteria. In this context, chitinase C from *S. griseus* HUT6037 was the first example of a bacterial member of family 19 chitinase that was identified [35]. Thus it is a good candidate to accomplish an evolutionary linkage between Actinobacteria and Viridiplantae [36,37].

The presence of a chitinase in grape extracts was confirmed by using acetazolamide, a well-known broad-spectrum chitinase inhibitor, which was able to inhibit the chitinolytic activity of both RGSM and *S. griseus* enzyme (Table 1). This is a remarkable result since it is well-known that members of the chitinase family 19 are heterogeneous. Although its inhibitory effect on RGSM extracts was low, acetazolamide resulted to be effective with both plant and bacterial enzymes. Accordingly, it can be suggested as a suitable inhibitor of chitinases from organisms of different kingdoms.

Aiming at better understanding why an Ab, raised toward a flavonoid binding sequence, can also recognize plant PR-proteins, the interaction among flavonoids and class IV chitinase was further investigated. In particular, the possible connection between chitinases and flavonoids was elucidated by assaying the effect of two of the most representative flavonoids present in grape, namely CA and QC on chitinase activity [12]. The functional assay was performed on both *S. griseus* chitinase and on a RGSM extracts containing class IV chitinase (supplementary Figure S1 and Figure 5). In either cases, a sort of hormetic effect was observed, thus indicating that flavonoids may actually act as modulators, rather than mere inhibitors, with a typical kinetics already observed in the case of other derivatives having pharmacological properties [38]. This effect was confirmed by two different assays, thus excluding any possible artefact; for sake of brevity, only one has been shown in this study. The functional comparison between the bacteria and RGSM chitinase showed that the modulation was strong and significant in the first case, while was less pronounced in the remaining one. This was ascribed to the flavonoids, already present in RGSM preparations, which probably acted as inner modulators, reducing any further possible measurable effect.

The results reported above on the modulation of the chitinase activity exerted by QC, CA and on the inhibition by anti-peptide Ab, let us to speculate that all these molecules may interact with the enzyme region devoted to binding of flavonoids. Moreover, the hormetic effects reveal that the enzyme has a conformational flexibility, controlled by flavonoid binding. Also in the case of the measurements of the effect of the anti-peptide Ab, on chitinase activity (Figure 4), experiments were performed by two independent assays to exclude any possible artefact. Since sequence comparisons with bilitranslocase (Table 2 and data not shown) have suggested that the site recognized by the anti-peptide Ab site is located in a highly accessible C-terminus region of both class IV and *S. griseus* chitinase, it is tempting to hypothesize that this structural portion may be involved in the interaction with flavonoids. In particular a biochemical analysis of kinetic features of the microbial chitinase (Figure 5 and Table 4) showed a hormetic effect both on KM and Vmax parameters, which were increased at low flavonoid doses, while higher treatments caused a decrease under the control level. The possible explanation about the hormetic behaviour of chitinase activity modulated by flavonoids is clearly merely speculative. As already claimed by Vargas and Burd [39], flavonoids, and quercetin in particular, show a biphasic effect on metabolic processes and catalytic activities depending on concentration, since at low concentration they act as reducing agents, while at high concentration they exert a pro-oxidative action. In the case of other auto-oxidizable molecules such as methylene blue, it has also been shown that their electron reduction-oxidation capacity was related to their in vitro hormetic dose-response modulation of the enzymatic activity [40]. Actually, there is evidence about redox status regulation on chitinolytic activity [41,42] and that plant chitinases and β-glucanases respond to UV-C treatment with a hormetic concentration-dependent manner [43].

All these modulatory effects showed by flavonoids support the dual action of these secondary metabolites, by having a complex regulatory role, depending on their concentration and redox state, in maintaining the homeostatic equilibrium among different cell metabolic processes. In addition, polyphenols often play also an ecological role as allelo-chemicals, being released in the environment to affect neighboring plant species. These physiological responses are themselves hormetic phenomena, which are described by complex mathematical models [44]. At the molecular level, hormesis probably results from the presence of multiple binding domains on the chitinase catalytic site, each of them characterized by a different binding affinity with both substrate and flavonoid. Such a complex interaction has already been proposed to explain hormetic response, for example in the case of mono-amine oxidase [45]. In our system, chitinase appeared to be not allosterically modulated, since the best regression curve for our data was a rectangular hyperbolic curve and not a sigmoid curve, as expected in the case of allosteric regulation.

In conclusion, the results reported in this study on the modulatory effect exerted by flavonoids on chitinase activity are in good agreement with recent literature, which evidenced that further physiological functions can be claimed for these secondary metabolites, in addition to the antioxidant and/or antimicrobial ones [6,46]. In fact, flavonoids have been demonstrated to act as developmental regulators and signals by a direct interaction with target proteins and modulation of activity [6,8,47]. Moreover, it has also been suggested that flavonoid localization in the nucleus may be associated to their role as activators/repressors of transcriptional factors [48].

During plant defence response to pathogen attack, flavonoid biosynthesis is induced in parallel with the activation of the expression of PR proteins, including chitinases. The results reported in this study on the modulation in vitro of chitinase activity by flavonoids, suggest that secondary metabolites may also exert such biological effects in vivo. These results may be useful for a better understanding of the intricate picture describing the various mechanisms underlying the plant response to biotic stresses. The modulatory effect of flavonoids on chitinase activity, together with PR protein expression, may be considered as an example of a regulatory convergence of two independent mechanisms of plant response to pathogens. This flavonoid property may also have practical consequences due to its possible impact on the augmentation of plant resilience. In fact, the use of flavonoids may be hypothesized to induce plant defence to pathogens by modulating the activity of PR-related proteins, in a similar way as other natural products regulate protective metabolic reactions/pathways [49,50]. Additional experiments have to be performed in this direction, together with a more detailed description of the structural basis regulating flavonoid-chitinase interaction.

## 4. Materials and Methods

### 4.1. Isolation of Microsomes from Berry Skin

Approximately 30 g (FW) of red grapevine (*Vitis vinifera* L., cv. Merlot) berry skin was homogenized as described in Braidot et al. [18], with minor changes: grape berries were pressed through 100 µm nylon gauze and the seeds were separated by floating into 10 mM Tris-HCl buffer at pH 7.5. The skins obtained were used to extract microsomes as described in the above-mentioned paper. For chitinase activity determination, microsomes were finally resuspended in 200 mM sodium acetate buffer, pH 5.0.

### 4.2. Anti-Peptide Antibody Production

Mice were immunized with a peptide corresponding to segment 235-246 (EFTYQLTSSPTC) of the primary structure of the mammalian bilitranslocase carrier [51]. Mouse antibody (Ab) was generated by cell fusion of mouse spleen lymphocytes and mouse myeloma NS1 cells, as described before [20]. Ab was purified from the growth medium by affinity FPLC on protein G column (General Electric, Healthcare, Little Chalfont, UK). Purified Ab was used for immunodetection at a concentration of 5 μg·IgM·mL^−1^.

### 4.3. SDS-PAGE and Subsequent Protein Extraction from Polyacrylamide Gel

Aiming at enriching the protein samples, 0.5 mg of protein from red grape skin microsomes (RGSM), conditioned in 62.5 mM Tris-HCl pH 6.8, 2.5% (*v*/*v*) SDS, 0.002% (*v*/*v*) bromophenol blue, 0.71 M β-mercaptoethanol, 10% (*v*/*v*) glycerol, were loaded onto a large SDS-PAGE 3 × 50 μL-volume well, with lateral wells for standard molecular markers. Proteins from RGSM were separated by SDS-PAGE (12%), as described in Braidot et al. [18]. After running, selected gel slices, corresponding to the band of interest able to cross react with antipeptide Ab (see also Section 4.5. Western Blotting), were cut and crushed in 4 mL of 25 mM Tris/250 mM glycine (Tris-glycine 1×). After centrifugation at 5000× *g* for 5 min, solubilized proteins were collected and concentrated using a Vivaspin filter (with 10 kDa molecular mass cut off), after 3 washing steps with 1 mL of Tris-glycine 1× to remove SDS.

### 4.4. Two-Dimensional Electrophoresis

Proteins recovered from SDS-PAGE were loaded on two-dimensional electrophoresis (2-DE); experiments were always performed in duplicate for further western blotting (WB) (30 µg protein loaded/strip) and silver staining analyses (3 µg protein loaded/strip). In the first dimension, isoelectric focusing of samples was performed on 3–11 NL strips (GE Healthcare), using an Ettan IPGphor 3 Isoelectric Focusing System unit (GE Healthcare), according to manufacturer’s instructions. Focused strips were equilibrated with dithiothreitol (DTT) and iodoacetamide, according to manufacturer’s instructions. Second dimension was performed by positioning the strips at the top of the running gels and performing SDS-PAGE as reported above (Section 4.3). After running, one gel was used for WB, which was carried out as reported below; the other gel was subjected to silver staining, as reported in Bortolussi et al. [52]. In both cases, gel images were acquired, analyzed and matched, as described by the same authors. Silver-stained gel spots corresponding to those detected by WB were excised, and further treated for protein identification.

### 4.5. Western Blotting

Gels from mono- and 2-D analyses were transferred onto nitrocellulose membranes and subjected to immunoblotting according to the protocol described in Braidot et al. [18]. In this case, the primary anti-peptide Ab (see the Section 4.2 on antibody production) was used at a final concentration of 5 µg·mL^−1^. Secondary antibody against mouse IgM (product A9044, Sigma Aldrich, Milan, Italy) was used at final dilution of 1:15,000.

### 4.6. Protein Identification

Gel spots of interest were triturated, washed with water, in-gel reduced with DTT, *S*-alkylated with iodoacetamide, and then in-gel digested with trypsin. Resulting peptide mixtures were desalted by μZip-TipC18 using 50% (*v*/*v*) acetonitrile and 5% (*v*/*v*) formic acid as eluents. Recovered peptides were then analyzed for protein identification by nano-liquid electrospray-linear ion trap-tandem mass spectrometry (nLC-ESI-LIT-MS/MS), using an LTQ XL mass spectrometer (Thermo Fisher Scientific, Waltham, MA, USA) equipped with a Proxeon nanospray source connected to an Easy-nanoLC (Proxeon, Odense, Denmark). Peptides were separated on an Easy C18 column (100 mm × 0.075 mm, 3 μm) (Proxeon). Mobile phases were 0.1% (*v*/*v*) formic acid (solvent A) and 0.1% (*v*/*v*) formic acid in acetonitrile (solvent B), running at a total flow rate of 300 nL·min^−1^. Linear gradient was initiated 20 min after sample loading; solvent B ramped from 5% to 35% over 45 min, from 35% to 60% over 10 min, and from 60% to 95% over 20 min. Spectra were acquired in the range *m*/*z* 400−2000. Peptide samples were analyzed under collision-induced dissociation (CID)-MS/MS data-dependent product ion scanning procedure, enabling dynamic exclusion (repeat count 1 and exclusion duration 60 s) over the three most abundant ions. Mass isolation window and collision energy were set to *m*/*z* 3 and 35%, respectively [53].

Raw data from nLC-ESI-LIT-MS/MS analysis were compared by MASCOT search engine (version 2.2.06, Matrix Science, London, UK) against a database containing protein sequences from *Vitis vinifera*, which were downloaded from the National Center for Biotechnology Information and UniProtKB database. Database searching was performed by using Cys carbamidomethylation and Met oxidation as fixed and variable modifications, respectively, a mass tolerance value of 1.8 Da for precursor ion and 0.8 Da for MS/MS fragments, trypsin as proteolytic enzyme, and a missed cleavage maximum value of 2. Other MASCOT parameters were kept as default. Protein candidates assigned on the basis of at least 2 sequenced peptides with an individual peptide expectation value <0.05 (corresponding to a confidence level for peptide identification >95%) were considered confidently identified. Definitive assignment was always associated with manual spectra visualization and verification.

### 4.7. Chitinase Activity Assays

Two different assays were used to evaluate the chitinase activity of both commercial enzyme from *Streptomyces griseus* (Sigma Aldrich) and RGSM preparation, following, respectively, the protocol of Magnin-Robert et al. [13] (data presented in Figure S1) and the protocol of Schuttelkopf et al. [23], with minor changes. Briefly, 2 µg of enzyme were mixed with the desired amount of the antibody in McIlvain’s buffer, pH 5.5 (100 µL-final volume) and pre-incubated in a black 96-well plate for 15 min (data presented in Figure 5 refer to the latter protocol). The fluorescent substrate 4-methylumbelliferyl β-d-*N*,*N*′,*N*′-tri-acetyl-chitotrioside (Sigma Aldrich) was dissolved in McIlvain’s buffer, pH 5.5, added to the mixture as substrate at final concentration of 0.5–100 µM, and then incubated at 37 °C, for 1 h. Finally, 100 µL of 1 M Na_2_CO_3_ was added to each well and the fluorescence was measured by means of a Multilabel Counter (WALLAC, model 1420, Perkin-Elmer, Waltham, MA, USA) set at 340 nm (20 nm excitation filter bandwidth) and at 465 nm (20 nm emission filter bandwidth). The same protocol was applied when 30 µg of RGSM was used instead of pure chitinase. For modulation experiments by the inhibitor acetazolamide, 1 µg of enzyme was pre-incubated for 30 min.

### 4.8. Statistical Data Analysis

All the experiments were carried out with at least three biological replicates, unless differently stated. In the case of chitinase activity determinations, treatment means were compared by Least Significant Difference (LSD), according to Fisher’s statistical test, and different letters assigned to means designate a statistical difference at *p* ≤ 0.05.

## 5. Conclusions

The identification of a chitinase in microsomes from grape berry skin was assessed by an antibody raised against a sequence of rat bilitranslocase. The antigen sequence of the mammalian protein is also a flavonoid binding domain. Consistently, the putative chitinase activity was found to be modulated by flavonoids. Such evidence was obtained in both grape extracts and pure commercial preparation from *S. griseus*.

These findings are noteworthy, because chitinase family plays a pivotal role as pathogen-related (PR) proteins, a class of enzymes involved in both plant responses to biotic and pollution stress, as well as in senescence. The modulation exerted by flavonoids on this activity opens new possibilities to increase plant resilience towards environmental strains. In particular, recent researches have been developed in the field of plant induced resistance, by means of activation of plant immune system. It has been demonstrated that treatments with peptones or chitosans are able to strengthen plant defense, acting as activators of PR protein. In this view, flavonoids could further stimulate the induced resistance, thus minimizing the use of pesticides. Actually, this is a critical issue that needs to be developed, in particular in the case of viticulture practices.

## Figures and Tables

**Table 1:** Grape berry skin proteins recognized by the anti-peptide Ab. Spot number, UniProtKB/NCBI accession, protein description, MASCOT score, theoretical Mr and pI values, matched and unique peptides observed by MS analysis and sequence coverage (%) are given.

Spot	UniProtKB/NCBI Accession	Protein Description	MASCOT Score	Theor. Mass (kDa)	Theor. pI	Matched Peptides	Unique Peptides	Protein Coverage (%)
A1	Q7XAU6_VITVI/33329392	Class IV chitinase [*V. vinifera*]	82	25.6	5.15	2	1	6.6
A2	Q7XAU6_VITVI/33329392	Class IV chitinase [*V. vinifera*]	298	25.6	5.15	6	4	24.2
A3	Q7XAU6_VITVI/33329392	Class IV chitinase [*V. vinifera*]	338	25.6	5.15	5	4	24.2
A4	Q7XAU6_VITVI/33329392	Class IV chitinase [*V. vinifera*]	358	25.6	5.15	8	4	24.2
B1	F6HLL9_VITVI/225441373	Glucan endo-1,3-β-glucosidase [*V. vinifera*]	227	33.3	7.06	10	3	17.8
B2	F6HLL9_VITVI/225441373	Glucan endo-1,3-β-glucosidase [*V. vinifera*]	278	33.3	7.06	24	5	28.7
B3	F6HLL9_VITVI/225441373	Glucan endo-1,3-β-glucosidase [*V. vinifera*]	476	33.3	7.06	24	8	49.4
C1	A5BV65_VITVI/147784332	Triose phosphate isomerase [*V. vinifera*]	190	27.2	6.35	6	4	15.4
C2	731394960	Vicilin-like antimicrobial peptides 2-3 [*V. vinifera*]	338	97.0	6.95	11	8	10.0
	A5BV65_VITVI/147784332	Triose phosphate isomerase [*V. vinifera*]	113	27.2	6.35	2	2	10.2
C3	731394960	Vicilin-like antimicrobial peptides 2-3 [*V. vinifera*]	254	97.0	6.95	5	5	10.7
C4	731394960	Vicilin-like antimicrobial peptides 2-3 [*V. vinifera*]	338	97.0	6.95	11	8	10.0
	A5BV65_VITVI/147784332	Triose phosphate isomerase [*V. vinifera*]	113	27.2	6.35	2	2	10.2

**Table 2:** Sequence alignment of proteins detected as reacting with the Ab raised against the peptide 235–246 of bilitranslocase. The rat enzyme is reported at the bottom of the each sequence alignment, while the target one is shown at the top.

Protein Description	Apparent Mass	Alignment	L-Align E Value
Class IV chitinase Accession n.: Q7XAU6	27.5 kDa 264 aa	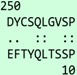	E < 0.29 60% similarity 40% identity
Chitinase (from *S. griseus*) Accession n.: WP_044369170	31.3 kDa 289 aa	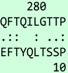	E < 0.13 70% similarity 40% identity
Glucan endo-1,3-β-glucosidase Accession n.: XP_002277446	36.7 kDa 340 aa	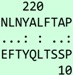	E < 0.18 80% similarity 30% identity
Triose phosphate isomerase Accession n.: CAN70587	27.2 kDa 254 aa	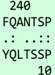	E < 0.046 86% similarity 43% identity
Vicilin-like antimicrobial peptides 2-3 Accession n.: XP_003632318	97.0 kDa 843 aa	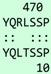	E < 0.14 71% similarity 71% identity

**Table 3:** Inhibition of the chitinase activity of RGSM and *S. griseus* by acetazolamide. The inhibitory effect of acetazolamide was tested according to the protocol of Schuttelkopf et al. [23].

	*S. griseus* (Fluorescence, A.U. h^−1^)	RGSM (Fluorescence, A.U. h^−1^)
Control	78,202 ± 3899 (100%)	40,397 ± 1654 (100%)
Acetazolamide (32 µM)	61,859 ± 2027 (79.1%)	34,091 ± 1152 (84.8%)

**Table 4:** QC and CA modulation of the chitinase activity of *S. griseus*. Chitinolytic activity by chitinase from *S. griseus* was measured using 4-methyl-umbelliferyl β triacetyl chitotrioside as a substrate, as described in the Material and Methods section. The enzyme activity was assayed in the absence and in the presence of different concentrations of QC and CA. The apparent Michaelis-Menten parameters were obtained at each substrate concentration, by fitting data to the Michaelis-Menten equation.

Flavonoid (µM)	K_M_ (µM) Quercetin	V_max_ (nmol 4-4-Methyl-umbelliferone (mg prot h)^−1^) Quercetin	R^2^	K_M_ (µM) Catechin	V_max_ (nmol 4-4-Methyl-umbelliferone (mg prot h)^−1^) Catechin	R^2^
0	35.42 ± 4.18	802 ± 41	0.996	48.89 ± 4.72	972 ± 46	0.998
0.5	42.05 ± 4.50	976 ± 48	0.997	68.81 ± 8.76	1190 ± 85	0.997
3	58.67 ± 2.91	1077 ± 28	0.999	78.33 ± 16.66	1183 ± 148	0.994
10	28.86 ± 4.94	598 ± 40	0.991	93.27 ± 7.60	1494 ± 76	0.999
20	28.70 ± 3.54	472 ± 23	0.995	53.21 ± 2.07	1007 ± 20	0.999

## Supplementary Materials

Supplementary materials can be accessed at: http://www.mdpi.com/1420-3049/21/10/1300/s1.
