# Supplementary material for: Flavonoid Interaction with a Chitinase from Grape Berry Skin: Protein Identification and Modulation of the Enzymatic Activity
Source: Molecules. 2016 Sep 28;21(10):1300. doi: 10.3390/molecules21101300 (PMC6273270; doi:10.3390/molecules21101300)
Supplement: Supplementary file 1 [file molecules-21-01300-s001.pdf]

Supplementary Materials: Flavonoid Interaction with a Chitinase from Grape Berry Skin: Protein Identification and Modulation of the Enzymatic Activity

Antonio Filippi, Elisa Petrussa, Uros Rajcevic, Vladka Čurin Šerbec, Sabina Passamonti, Giovanni Renzone, Andrea Scaloni, Marco Zancani, Angelo Vianello and Enrico Braidot

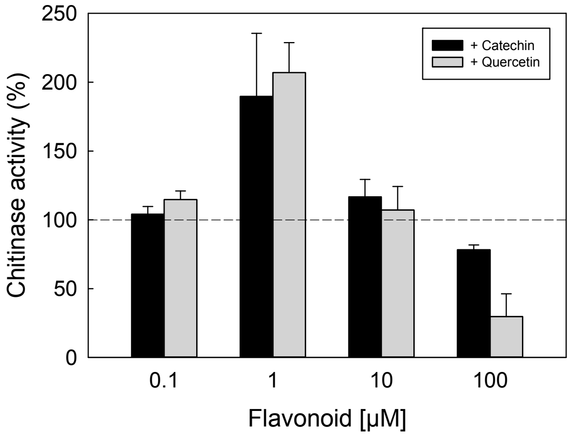

Figure S1. QC and CA modulation of the chitinase activity of *S. griseus*. Chitinolytic activity was measured as described in Magnin-Robert et al. [13]. Different concentrations of CA (black bars) and QC (grey bars) were tested on chitinase from *S. griseus*. The values are expressed as percentage respect to the control, represented by the 100% of activity (dashed line).
